# Medical Topography of Upper Canada

**Published:** 1819-10-01

**Authors:** 


### *Medical Topography of Upper Canada.*

By John Douglas, Assistant Surgeon, 8th Regiment. Octavo, pp. 126. London, 1819.

When the English traveller views the extent to which our arts and our arms have spread over the surface of this globe, he may well cry out, in the language of Æneas,—

> " Quæ Regio in terris nostri non plena laboris?"

War as well as commerce enlarges the boundaries of science—especially of medical science; but it is in the bosom of Peace that we can best arrange and combine those scattered fragments of knowledge, that have been gleaned in bloody fields, toilsome marches, and tedious voyages. The period is arrived when the gate of Janus is closed, and the temple of Science opened to receive the offerings of her sons, and honourably record their names. The medical non-combatants of our fleets and armies, though they shared equally in the toils, did not reap equally the honours of warfare. It is but justice, therefore, that they should *now* come in for their portion of laurels, dearly and honestly earned, it is true, but, on that account, more imperishable in their nature, and unfading in their bloom.

During the late unhappy contest between two nations, that once were linked in the bonds of consanguinity, and still are united by the golden chain of commerce, our soldiers were widely distributed over those districts of Upper Canada, which immediately border upon the United States. The appearances which Nature presents to the eye in this part of the globe are sublime and interesting. From the declivities of lofty mountains, immense rivers and mighty streams descend in various directions to the ocean, traversing lakes of vast extent, and precipitating themselves over chains of rocks in the form of roaring cataracts. Human art has never been employed here to perpetuate the revolutions of the past. Nature is the only record.

The habitual tranquillity which the Settlers of Canada had long enjoyed, was much interrupted by the tumult of war. Fathers and sons renounced for a season their social relations, to arm in defence of their province. While hostilities were carrying on, their houses were at times pillaged and burnt down by the enemy; their fences and orchards destroyed; their fields laid waste; mothers flying in the dead of night, with their children in their arms, while their dwellings were seen at a distance in flames!

The medium heat of the summer, along the southern boundaries of the province, is near to a tropical scale; the enclosed lands being exposed to the sun, while the breezes are intercepted by lofty forests. A beautiful vegetation clothes those plains which were lately sheeted over with water; but from these an offensive odour is, at times, evolved from the soil, along with the copious evaporation of moisture. The sun, thus operating on a humid ground, and completing the decomposition of great masses of organic remains, tends to the production of those diseases which assail the unassimilated constitution.

In spring, catarrh and pneumonia were frequent, both dependent on atmospherical vicissitudes. In some men the symptoms of pneumonia assumed a very unusual appearance, the marks indicative of the disease being very obscure. "The morbid action of the pulmonary vessels seemed to be smothered." The pulse was but little accelerated; in a few instances, low and depressed; the heat of skin not much increased; the patients often complaining of listlessness and debility, rather than of pain in any part of the chest. "By the abstraction of blood the character of the disease was soon made manifest." Sir James M'Gregor, in his interesting account of the diseases which prevailed in the peninsular army, has delineated the peculiar features of this obscure and dangerous form of thoracic inflammation, with much force.

Mr. Douglas found it necessary to carry depletion to a great extent, in order to combat pneumonia in all forms. If blood was not freely abstracted within the first thirty-six hours of the attack, fatal effusion in the lungs was the usual result. The appearances of the blood and the pain in respiration were the sure indications for venesection.

"It is a remarkable fact, that in the early stage of inflammation, death has never directly followed the abstraction of a large quantity of blood."

A consideration which, he thinks, ought to inspire confidence in the mind of the young practitioner.

Acute rheumatism, like pneumonia, derived its origin from the sudden transitions of heat and cold. In some patients, affections of the liver, and a general yellowness of the skin were attendant on the complaint.

"After the inflammatory symptoms had yielded to the usual mode of treatment, the lower extremities often became œdematous. Those cases of the disease, combined with obstructed or diseased viscera, were much benefited by mercurial salivation." 61.

Cholera morbus was occasionally met with on a march, and was often attributable to sudden changes of temperature.

In the summer and autumn of 1813, the remittent, or, as it is there called, the Lake Fever, prevailed to some extent, deriving its origin from vegeto-animal exhalations, as usual. The symptoms were the same as have been described by all late writers; and the treatment, on its first attack, consisted chiefly in depletion, both general and local.

"Calomel and jalap were the principal purgatives that were used. When delirium or coma supervened, blisters were applied to the head, and calomel, combined with James's powder, was given in such quantities as might affect the mouth."

Mr. Griffith, of the Royals, observes, in a letter to the author,

"I bled freely in Canada, but not so plentifully as within the tropics. In the remittent of Canada, the ablution of the body with cold water often brought on a remission, after which the bark was given with advantage. Calomel, however, was the principal remedy; for, when the mouth became affected with that medicine, the patient had, for the most part, a speedy recovery, and was not so liable as others who were treated in a different manner, to be attacked with intermittent fever."

In respect to dysentery, our author found gentle bleedings, a few purgatives, and attention to diet, generally overcome the complaint. Chronic dysentery he considers as connected with derangement of the liver or its secretions—"for the liver was observed diseased in a number of patients who have fallen victims to this form of the complaint."

"Mercurial friction, when it is pushed so far as to affect the mouth, almost always suspends the dysenteric symptoms."

Mr. Douglas observed, that immediate amputation after wounds was much more fortunate in its results, than where procrastination was used. No case of tetanus occurred in Canada.

Upon the whole, we read this little volume with much interest, and consider that it is indicative of both zeal and merit in the author. It contains many medico-topographical remarks which may prove serviceable to the army surgeon about to embark for our transatlantic colonies of the north; and it is indeed worthy of a careful perusal by the medical officers of both services.

